# Editorial: Functional nutritional strategies as alternatives to antimicrobials

**DOI:** 10.3389/fvets.2025.1711519

**Published:** 2025-10-20

**Authors:** Matteo Dell'Anno, Monika Hejna, Francesca Riva, Luciana Rossi

**Affiliations:** ^1^Department of Veterinary Medicine and Animal Sciences—(DIVAS), University of Milan, Lodi, Italy; ^2^Department of Veterinary Sciences, University of Messina, Messina, Italy; ^3^Department of Bacterial Physiology, Institute of Microbiology, Faculty of Biology, University of Warsaw, Warsaw, Poland; ^4^School of Biodiversity, One Health and Veterinary Medicine, University of Glasgow, Glasgow, United Kingdom

**Keywords:** antimicrobial resistance, phytogenics, natural compounds, functional diet, livestock, pet, gut health, one health

The challenge of antimicrobial resistance (AMR) has emerged as one of the major concerns within the One Health perspective, and it is currently being addressed at multiple levels by international organizations, policymakers, and the scientific community. Important efforts focus on both human and animal health, promoting the judicious use of antimicrobials and encouraging the development of sustainable alternatives to safeguard public health, animal welfare, and environmental sustainability. In particular, over the past few decades, scientific research has increasingly focused on exploring innovative nutritional interventions as functional strategies to enhance disease prevention, promote overall animal health, reduce the need for antibiotics, and mitigate the spread of AMR.

This Research Topic includes 19 contributions focused on the potential of functional nutritional strategies as promising alternatives to antibiotic treatments for both livestock and companion animals. Several articles in this Research Topic explore the roles of probiotics, prebiotics, postbiotics, and natural and plant-derived compounds in modulating the microbiome, immune responses, and metabolic pathways. These studies highlight how dietary supplementation can strengthen host defenses, mitigate oxidative stress, and reduce the incidence of disease conditions.

Taken together, the contributions to this Research Topic on swine highlight the multi-mechanistic modes of action of functional compounds, encompassing anti-inflammatory and antioxidant effects, enhancement of barrier function, and modulation of the microbiome and metabolome axis. Several nutritional strategies have been investigated to mitigate weaning stress, shape the gut microbiota and metabolite profiles, and ultimately improve animal performance and health. For instance, microencapsulated essential oils were shown to support both health and performance in weaned piglets (Xu et al.), while essential oil from *Alpinia oxyphylla* modulated immunity, antioxidant capacity, and the gut microbiota profile in fattening pigs (Chen et al.). Probiotic supplementation increased beneficial short-chain fatty acids in the feces and improved average daily gain in weaned piglets (Zhao et al.). Another study on piglets demonstrated that oral supplementation with an egg yolk immunoglobulin-based product improved growth performance and mucosal immunity, which was particularly beneficial for low-birth-weight neonates as it enhanced β-defensin expression, modulated inflammatory responses, and stabilized intestinal barrier function during the pre-weaning period (Lothong et al.). Maternal nutrition has also emerged as a critical determinant of offspring resilience. Fang et al. showed that perinatal fish oil supplementation in sows protected piglets from LPS-induced damage, while Daneshi et al. revealed that maternal one-carbon metabolite supplementation modulated the expression of antimicrobial peptides in both fetal and maternal tissues, suggesting an epigenetic route through which perinatal nutrition can shape innate immunity.

Plant-derived polyphenols also confirmed their beneficial properties. Supplementation with *Ampelopsis grossedentata* extract improved intestinal morphology, enhanced antioxidant status, and modulated the gut microbiota in growing-finishing pigs (Liu, Zhang et al.). Two complementary studies focused on seaweed supplementation. The first included an *in vivo* trial with post-weaning piglets, demonstrating that a blend of *Ascophyllum nodosum* and *Phymatolithon calcareum* protected against F4+ *E. coli* infection by improving gut morphology and antioxidant status (Dell'Anno et al.), while a second companion *in vitro* characterization and zootechnical trial confirmed that the bioactive compounds retained their functionality after simulated digestion, indicating their synergistic antioxidant and antimicrobial properties (Frazzini et al.). A dedicated review highlighted the importance of lactoferrin as a multifunctional protein and an alternative antimicrobial compound. Beyond its well-documented immunomodulatory and antimicrobial properties, the authors focused on its proteolytic activity, which can impair bacterial virulence factors and decrease pathogen colonization. While the exact mechanisms are not always clear, the proteolytic feature of lactoferrin could represent a valuable avenue for limiting pathogenicity and tackling multidrug-resistant bacteria (Ongena et al.).

Similarly, in broilers, a synbiotic enzyme complex (mannose oligosaccharides, *Clostridium butyricum, Bacillus subtilis*, glucose oxidase, and α-galactosidase) improved growth, carcass traits, immune organ indices, and intestinal histomorphology compared to an antibiotic-treated control group (Zhao et al.). Similarly, dietary inclusion of a *Bacillus subtilis*-derived postbiotic enhanced performance and reduced mortality rates in broiler chicks (Li et al.). Furthermore, the evaluation of an antimicrobial peptide derived from the Isalo scorpion demonstrated a beneficial effect on growth performance, immune function, and gut microbiota in yellow-feathered broilers (Wang et al.), underscoring the value of natural bioactive compounds as alternatives to antibiotics.

Studies on ruminants in this Research Topic emphasized both direct and maternal nutritional pathways. A trial with *Saccharomyces cerevisiae* strain KA500 supplemented in the feedlot buffalo's diet revealed a trend toward increased daily weight gain (Ferreira et al.). In a cattle study, maternal nutrition and neonatal antimicrobial peptide responses Daneshi et al. highlighted how early-life immune competence may be “nutritionally primed”. Another study investigated a promising medium-chain fatty acid combination used as silage additives, which reduced undesirable fermentations and pathogenic risks in corn silage (Salinas-Chavira et al.), helping to mitigate contamination pressure before it reaches the animal. In weaned yaks, traditional Chinese medicine-based compounds were shown to enhance physical fitness, antioxidant capacity, and immune status under high-altitude conditions (Lu et al.), underlining how local functional solutions can further align with precision nutrition strategies, along with general sustainability.

Beyond terrestrial systems, supplementation with low-dose dietary arabinoxylan (< 5%) was well tolerated in rainbow trout, whereas high-dose supplementation (10%) impaired intestinal barrier function, altered gut microbiota, and reduced antioxidant capacity and protein metabolism, thereby clarifying the anti-nutritional effects of arabinoxylan and supporting adjustments in plant-based aquaculture feed formulation (Zhang et al.). Given the rapid growth of aquaculture and its specific antimicrobial demand challenges, the optimization of functional ingredient feed formulas offers scalable solutions that align well with large-scale feed production.

Furthermore, two studies emphasized the potential of probiotic and prebiotic strategies in companion animals. Lactic acid bacterial strains isolated from canine feces were characterized as promising probiotic candidates (Liu, Wang et al.), while a trial revealed that a prebiotic fiber blend enhanced fecal quality and raised the concentrations of short-chain fatty acids in the feces of puppies (McGrath et al.).

Across species, several key themes emerged: (i) improvements in growth or feed efficiency are often associated with changes in gut morphology, immune function, and antioxidant status; (ii) shifts in microbiota and metabolite profiles may be crucial for unraveling the mechanisms of action of functional compounds; and (iii) algae, plant polyphenols, essential oils, and pro-, pre-, and post-biotics offer complementary strategies that can also be used in combination.

In conclusion, the studies included in this Research Topic demonstrate that functional nutritional strategies can significantly enhance animal health and performance while reducing the need for antibiotic treatments. The variety of approaches and species emphasizes the broad applicability of these strategies.

As demonstrated by these studies, reducing antibiotic use fundamentally relies on promoting animal health. Alternatives to antibiotics encompass all strategies aimed at minimizing antibiotic administration. Ensuring good health in animals reduces the need for antibiotics, thereby lowering the selective pressure for antimicrobial resistance and limiting the dissemination of resistance genes among bacterial populations in animals, humans, and the environment, including both pathogenic and commensal strains.

